# Comparison of Physical Aging and Glass Transition in Glassy–Rubbery Polymer Bilayer Films

**DOI:** 10.1021/acs.jpcb.4c07902

**Published:** 2025-03-03

**Authors:** Jennifer A. McGuire, James H. Merrill, Alexander A. Couturier, Michael F. Thees, Connie B. Roth

**Affiliations:** Department of Physics, Emory University, Atlanta, Georgia 30322, United States

## Abstract

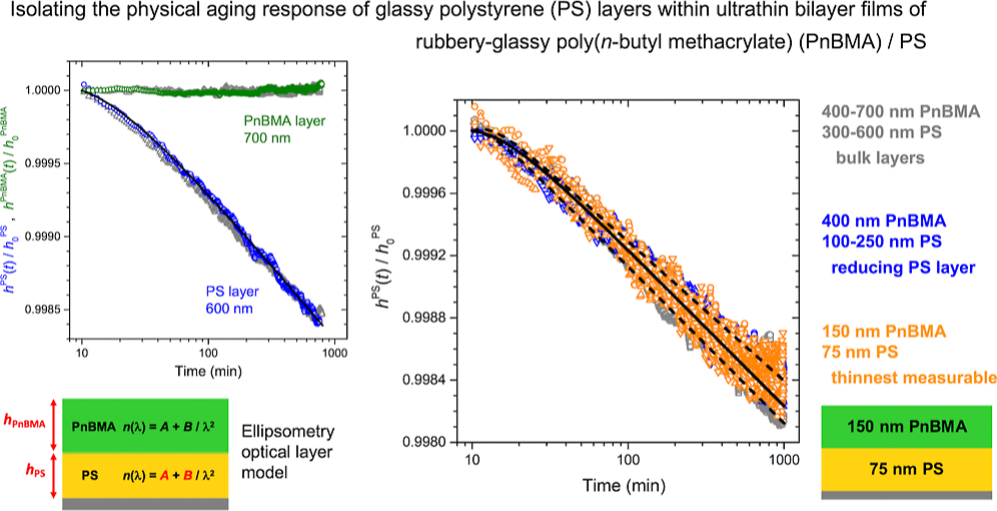

In the present work, we use ellipsometry to extract the physical aging response of thin glassy polystyrene (PS) layers from rubbery–glassy bilayer films of poly(*n*-butyl methacrylate) (PnBMA) atop PS. How the soft interface between rubbery and glassy polymer domains can impact the physical aging response of glassy domains is unclear. Measurements in the literature have shown that the local glass transition temperature *T*_g_ of PS is strongly reduced near a PnBMA/PS interface with a magnitude twice as large compared to that imparted by a free surface. As the free surface is known to reduce physical aging, we anticipated large changes in the physical aging response of PS within PnBMA/PS bilayer films. However, surprisingly the aging response remained equivalent to bulk down to 75 nm PS layer thicknesses that were the thinnest we found could be accurately measured given the optical limits of dispersion. With complementary fluorescence measurements, we show that the average *T*_g_(*h*_PS_) of such PS layers within 150 nm PnBMA/75 nm PS bilayer films are also still bulk. These findings demonstrate that films with finite domain sizes have interfacial dynamical gradients that are significantly altered from those previously measured in systems with semi-infinite domain sizes.

## 1 Introduction

Interfacial effects are known to strongly impact the properties of thin films and other material geometries with nanoscopic domain sizes, including physical aging.^1−14^ The presence of the interface typically leads to a perturbation locally that propagates into the material over some distance imposing a gradient in local properties.^5,7,8,15−21^ An important open question in the field currently is the degree to which different material properties are locally correlated.^5,18,19,22^ Are different properties such as the glass transition, physical aging, viscosity and modulus that are related in bulk, still related in the same manner locally in such confined systems? In the present work, we are interested in understanding how a rubbery–glassy polymer–polymer interface impacts the physical aging response of glassy layers. Physical aging corresponds to the long-term property changes that occur during structural relaxation of the nonequilibrium glassy state.^23−25^ Although the decrease in free volume associated with this structural recovery is tiny, typically only ∼0.5%, this can result in significant changes in dynamics and other material properties as the properties of glasses are highly sensitive to molecular packing.^23,26,27^ This long-time evolution of material properties can often be the limiting factor in applications as the stability of the glassy state can become compromised with outsized changes in modulus, brittleness, and factors such as gas permeability.^24,28^ The extent to which an interface between a rubbery “soft” polymer domain can impact the resulting stability and physical aging of a glassy “hard” polymer domain is still unclear and has important implications for a range of applications.^14,28−37^

The impact of the free surface has been the most heavily studied interfacial effect on the physical aging of thin glassy polymer layers. For polystyrene (PS) films, our present focus, reduced physical aging rates have been observed that appear to correlate reasonably well with decreases in the glass transition temperature *T*_g_ resulting from the free surface.^5,7,8^ The decrease in physical aging rate with decreasing film thickness *h* observed for supported PS films is not consistent with a simple shift in the film average *T*_g_(*h*).^5^ Instead, an analysis of the temperature dependence of the physical aging rate β(*T*) in thin films is reasonably consistent with a numerical average of a locally varying depth-dependent aging rate β(*z*) that would be anticipated given a known locally varying depth-dependent *T*_g_(*z*).^5,7,8^ These studies suggest a local correlation between physical aging and *T*_g_, as was demonstrated for poly(methyl methacrylate) (PMMA) thin films by localized fluorescence measurements.^19^ Unfortunately, such a local probe is not available for PS. It is worth noting that these studies are a measure of the volumetric physical aging response, typically measured by ellipsometry, while the enthalpic physical aging response as measured by differential scanning calorimetry (DSC) appears to show slightly faster aging behavior in thin films relative to bulk.^38^ These results are puzzling, although it is known that volumetric and enthalpic physical aging rates are not necessarily correlated in bulk systems for a given polymer.^23^

In the present work we are interested in understanding what impact a rubbery–glassy polymer–polymer interface might have on the volumetric physical aging response of glassy PS films. Recent fluorescence measurements have demonstrated that rubbery–glassy polymer–polymer interfaces can have large and long-range perturbations to the local glass transition temperature *T*_g_(*z*).^18,20,39−41^ These studies on semi-infinite systems would suggest that a rubbery–glassy polymer interface causes a stronger perturbation to local dynamics than a free surface. For example, the local *T*_g_(*z*) in PS next to a domain of rubbery poly(*n*-butyl methacrylate) (PnBMA) has a local *T*_g_ reduction of ≈60 K,^20^ in comparison to that near a free surface of ≈30 K.^15^ The local *T*_g_(*z*) also extends much further into the PS domain from the interface, ≈250 nm from the PnBMA/PS interface in comparison to ≈25 nm from a PS free surface.^15,18,20^

Prior to these studies, we investigated the physical aging rate of glassy PS layers atop bulk PnBMA layers.^29^ This work demonstrated that ellipsometry data of PS/PnBMA bilayers could be sufficiently well resolved to separately extract the layer thickness of the PS and PnBMA layers. However, the geometry of these samples with thin PS layers atop bulk ∼500 nm PnBMA layers left the resulting conclusions ambiguous because both the presence of the top free surface and the underlying PS/PnBMA interface could be perturbing the PS layer. Surprisingly, no change in the physical aging rate of the glassy PS layer relative to bulk was observed down to PS layer thicknesses of 84 nm, the thinnest measured, although with relatively large error bars for the aging rate of the thinnest films.^29^ At this thickness, the PS layer *T*_g_ was strongly reduced by ≈25 K relative to bulk, such that it was anticipated both interfaces would act to reduce the physical aging rate of the thin glassy PS layer. As this was not the case, questions remained regarding the influence of the rubbery–glassy interface and whether some alternate factor such as local plasticization or stress relaxation was at play.

In the present work, we revisit the PS and PnBMA bilayer system, but with an important change to the sample geometry that allows us to isolate the impact of the rubbery–glassy PnBMA/PS interface. Ellipsometry measurements are performed on PnBMA/PS bilayer films where the thin glassy PS layer is first spin-coated on the silicon substrate as the bottom layer followed by floating a rubbery PnBMA layer atop to cap the PS layer. This sample geometry is most akin to the supported PS films that deduced the impact of the free surface on the PS physical aging response.^5,7,8^ The underlying SiO_*x*_–Si interface of silicon substrates with native oxide layers do not appear to alter the physical aging rate as they do not perturb the local *T*_g_.^15,42−44^ With this PnBMA/PS/SiO_*x*_–Si sample geometry only the top interface of the glassy PS layer, in this case the rubbery–glassy PnBMA/PS interface, is expected to influence the physical aging response of PS. In the present measurements, we have extended the aging time out to 1000 min in order to better characterize the physical aging response. We have also made improvements to the experimental procedures and ellipsometric optical layer model fitting that allows us to measure thinner layers, although we are ultimately limited by the wavelength of light in adequately resolving the dispersion in very thin bilayer films. Our findings surprisingly show no change in the physical aging response of thin glassy PS layers down to 75 nm in thickness, which coupled with complementary fluorescence *T*_g_ measurements, suggest that the overall domain size strongly alters the magnitude and breadth of the gradient in interfacially perturbed dynamics.

## 2 Experimental Methods

Bilayer samples for physical aging measurements were prepared by spin-coating PS (*M*_w_ = 1920 kg/mol, *M*_w_/*M*_n_ = 1.26, Pressure Chemical) onto 2 cm × 2 cm silicon wafers with a 1.25 nm native oxide layer (SiO_*x*_–Si).^45^ PnBMA (*M*_w_ = 319 kg/mol, *M*_w_/*M*_n_ = 2.6, Scientific Polymer Products) was spin-coated onto freshly cleaved mica. All bilayer samples were annealed following one of two protocols. The first protocol annealed the PS films spin-coated on silicon at 120 °C and the PnBMA films spin-coated on mica at 70 °C, separately under vacuum for 18–24 h. After annealing, bilayer films were assembled by floating the PnBMA film onto the PS/SiO_*x*_–Si sample using a water transfer procedure, then allowed to dry for at least 30 min. The second protocol assembled the bilayer films first via floating prior to annealing. Following bilayer assembly, the entire sample was annealed at 80 °C under vacuum for 18–24 h. Both protocols did a final annealing step of the entire bilayer on the ellipsometer hot stage (Instec HSC 302) at 120 °C for 30 min to erase thermal history and create a well-defined PnBMA/PS interface. Both protocols produced equivalent results.

**Figure 1:**
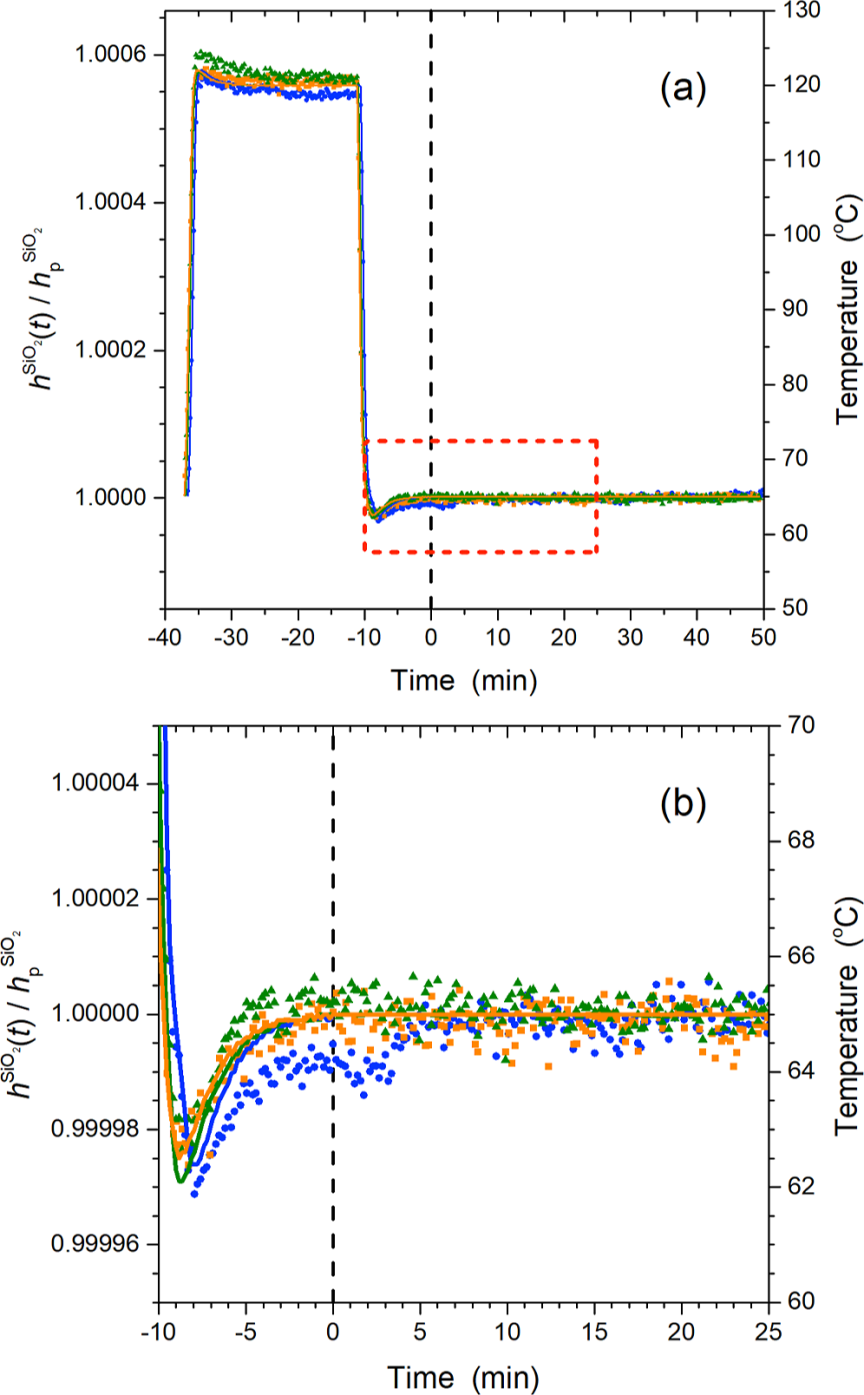
(a) Instrument stability verified by monitoring the thermal expansion response of a 1000 nm SiO_2_ thermal oxide layer during the temperature quench protocol used to initiate physical aging measurements. Solid curves show the temperature profile executed by the heater, a rapid heat and thermal equilibration at 120 °C followed by a fast (55 °C/min) temperature quench to the desired aging temperature. Symbols represent the thickness of the SiO_2_ thermal oxide layer *h*_SiO_2__(*t*), normalized to the final plateau thickness  reached by the sample at 65 °C. Three different runs demonstrate reproducibility. (b) Close up view of red box in (a) isolating the region when the sample reaches the aging temperature of 65 °C, used to define *t* = 0 (vertical dashed line).

Physical aging measurements using spectroscopic ellipsometry began with this final annealing step of 120 °C for 30 min on the ellipsometer (J. A. Woollam M-2000), followed by a rapid quench down to the desired aging temperature of 65 °C, at a cooling rate of 55 °C/min using the liquid-nitrogen capability of the Instec hot stage,^7^ where the samples were then held for 800–1000 min. Instrument stability to this rapid thermal quench on the ellipsometer Instec hot stage was verified by monitoring the thermal expansion response of a 1000 nm SiO_2_ thermal oxide layer on silicon (SiO_2_/Si). Because SiO_2_ has an extremely high glass transition temperature, compared to the protocol temperature range (20–120 °C), the sample responds almost instantaneously to changes in temperature and experiences no physical aging. Figure 1 demonstrates this by graphing the temperature profile carried out by the Instec hot stage during the rapid heat, subsequent equilibration at 120 °C, and then fast thermal quench to a temperature of 65 °C. Data are shown for the film thickness of the SiO_2_ thermal oxide layer as it expands and contracts in response to these temperature jumps, where solid curves denote the temperature profile of the heater. Multiple runs are shown to demonstrate reproducibility, where the SiO_2_ thermal oxide thickness has been normalized to the final plateau thickness *h*_p_ reached by the sample at 65 °C. The data in Figure 1b illustrates that the instrument stability for film thickness measurements is within 0.001%. Data shown are focused on the time around the temperature quench, however, runs have also been previously carried out for the entire aging protocol out to 1000 min to verify temperature and instrument stability for the long aging measurements. From these stability tests we defined *t* = 0 (vertical dashed line in Figure 1) for the aging measurements as the time at which the sample temperature reached and maintained the desired aging temperature of 65 °C.

**Figure 2:**
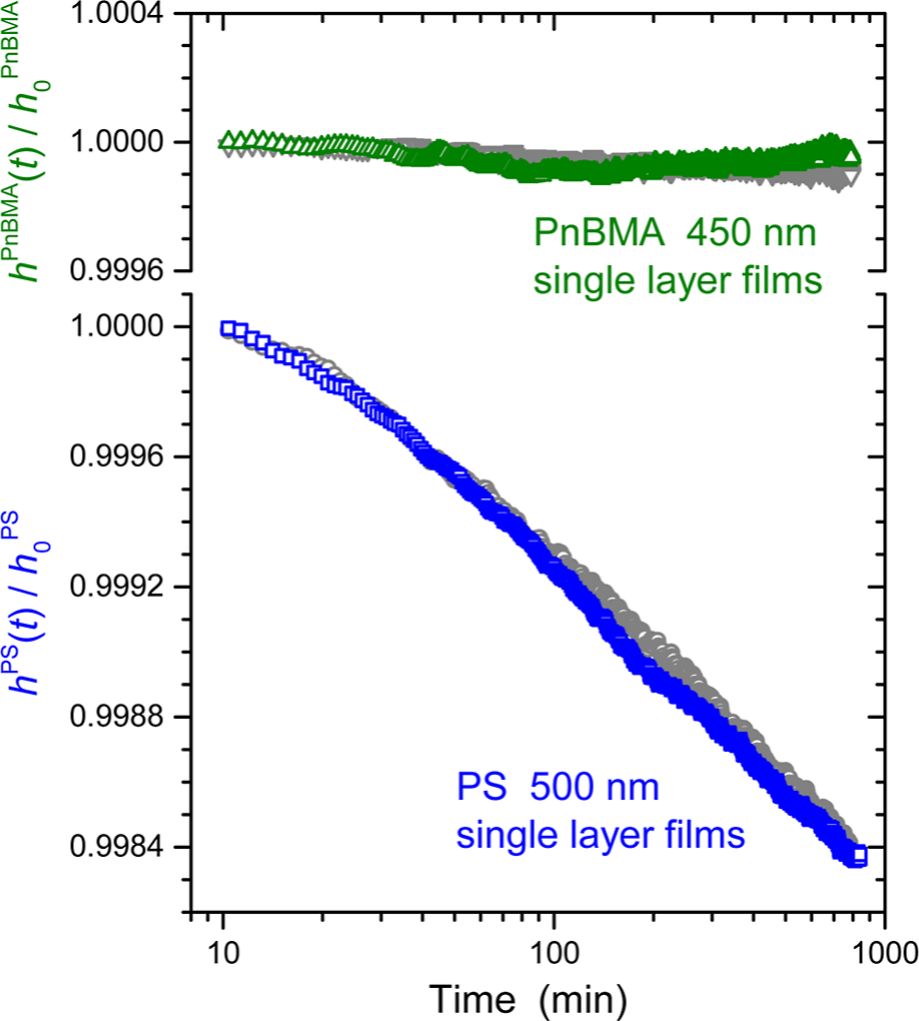
Single layer films of bulk PnBMA (top) and bulk PS (bottom) held at 65 °C following a temperature quench from 120 °C. Two data sets are shown for each type of film to demonstrate reproducibility. The range of the *y*-axes have been adjusted to make the scales the same illustrating that the PnBMA films (*T*_g_^PnBMA^ = 20 °C) maintain a constant thickness at this temperature, while PS films (*T*_g_^PS^ = 100 °C) show the decrease in *h*(*t*)/*h*_0_ on a log scale characteristic of physical aging.

Stability tests were also performed on single layer PnBMA films to characterize how the PnBMA layer would respond to the same temperature quench protocol. As in our previous measurements,^29^ dry nitrogen gas was flowed continuously through the sample chamber to minimize any uptake of water by the PnBMA layers. Bulk PnBMA films (≈450 nm) were spin-coated on silicon and vacuum annealed at 70 °C for 18–24 h. These films were then subjected to the same temperature protocol on the ellipsometer that are used for the aging runs (as depicted Figure 1). Similarly, bulk PS films (≈500 nm) on silicon, vacuum annealed at 120 °C for 18–24 h, were also investigated. Following a standard aging measurement, Ψ(λ) and Δ(λ) data were collected for 56 s every 120 s, with data for λ = 400–1000 nm fit to an appropriate layer model, a Cauchy layer *n*(λ) = *A* + *B*/λ^2^, atop a silicon substrate with 1.25 nm native oxide layer.^45^ Following our previous work,^7,46^ film thickness values are normalized to the thickness *h*_0_ corresponding to an aging time of 10 min, determined from an average of the thickness data over a 10 min period about this value. All aging data were smoothed using 5-point adjacent averaging. Figure 2 graphs the normalized thickness *h*(*t*)/*h*_0_ as a function of time for PnBMA and PS films held at 65 °C plotted on semilog axes, where the *y*-axes ranges have been adjusted to show the two films on the same scale. At 65 °C, PS films (*T*_g_^PS^ = 100 °C) demonstrate the characteristic decrease in *h*(*t*)/*h*_0_ on a log scale indicative of physical aging, while PnBMA films (*T*_g_^PnBMA^ = 20 °C) maintain a constant thickness at this temperature, as expected for a film in equilibrium. Even though the decrease in PS film thickness with aging time is small, ∼0.15%, it is significantly larger than the variability in the data and well above the instrument stability. Data from these single layer PnBMA samples were used to establish bulk PnBMA refractive index parameters of *A* = 1.456 and *B* = 0.00461 for the Cauchy model used for the PnBMA layers within the PnBMA/PS bilayers. (Note we have followed the common convention used within the Woollam software to quote values for the *A* and *B* Cauchy equation parameters with the wavelength λ calculated in microns.^45^)

Corresponding bilayer samples for the fluorescence measurements were constructed by using a low-label-content (0.34 mol %) pyrene-labeled PS (*M*_w_ = 582 kg/mol, *M*_w_/*M*_n_ = 1.58), synthesized by copolymerization of styrene with trace amounts of 1-pyrenylbutyl methacrylate.^29^ The same PnBMA was used. Both PS and PnBMA layers were spin-coated onto freshly cleaved mica substrates, and individually annealed under vacuum for ≈18 h at 120 °C for PS and 80 °C for PnBMA films. The PS layers were floated off the mica substrate and transferred onto quartz slides using filtered, deionized water. The PnBMA layers were then floated and transferred onto the top of the PS layers. The fully assembled bilayers underwent final annealing at 120 °C for 30 min on the fluorometer hot stage prior to the *T*_g_ measurements to ensure the PS/PnBMA interface was annealed to equilibrium.^39^

Fluorescence measurements in order to determine the *T*_g_ of the PS layer were performed using a Photon Technology International QuantaMaster spectrofluorometer with samples mounted in an Instec HCS 402 heater. Fluorescence intensity was measured for 3 s as a function of temperature starting every 30 s while cooling from above *T*_g_ at a cooling rate of 1 °C/min. The emission wavelength at 379 nm for the *T*_g_ measurement was chosen to be on the first emission peak identified by measuring the emission spectrum prior to each measurement of *T*_g_ at the high temperature (120 °C), in accordance with the procedure used in previous studies by our group.^29,44^ An emission spectrum was also collected after each measurement, reheated to the high temperature to check for excessive oxidative photobleaching. The *T*_g_ value was determined from the temperature-dependent intensity traces by linearly fitting the temperature-dependent slopes associated with the glassy and liquid regimes, and identifying their intersection point.^15,29^

## 3 Results and Discussion

### 3.1 Physical Aging of PnBMA/PS Bilayer Films

A key difference in the present measurements from that of our previous work^29^ is that the glassy PS layer whose physical aging is being investigated is sandwiched between the PnBMA layer at the top interface and the underlying SiO_*x*_–Si substrate at the bottom interface. This geometry removes the complications of competing free surface effects to the PS layer, as was present in our previous work with PS atop PnBMA. In the present geometry we anticipate that only the rubbery–glassy PnBMA/PS interface will act to perturb the aging dynamics of the PS layer, as previous studies have demonstrated that the PS/SiO_2_ interface is “neutral” and does not perturb the local *T*_g_,^15,42−44^ nor appear to perturb local physical aging.^5,7,8^ We are interested in determining how the presence of the PnBMA/PS interface alters the local dynamics and physical aging response of the PS layer, which means we aim to identify changes in the physical aging rate of the PS layer with decreasing PS layer thickness.

Bilayer samples of a PnBMA layer atop a PS layer supported on SiO_*x*_–Si wafers were fabricated and annealed at 120 °C for 30 min, sufficiently high above the bulk *T*_g_ of both layers to ensure that the PnBMA/PS interface had reached its equilibrium width of 7 nm for these high molecular weight polymers.^20,29,39,47^ The films where then thermally quenched at a rate of 55 °C/min to an aging temperature of 65 °C, corresponding to the peak in the aging rate for PS.^5,46^ Film thickness and refractive index changes of the sample are then monitored for an aging time of 800–1000 min. At 65 °C, the PS layer is in its glassy state (*T*_g_^bulk^ = 100 °C for PS), while the PnBMA layer is still in the equilibrium state above its *T*_g_ (*T*_g_^bulk^ = 20 °C for PnBMA).

**Figure 3:**
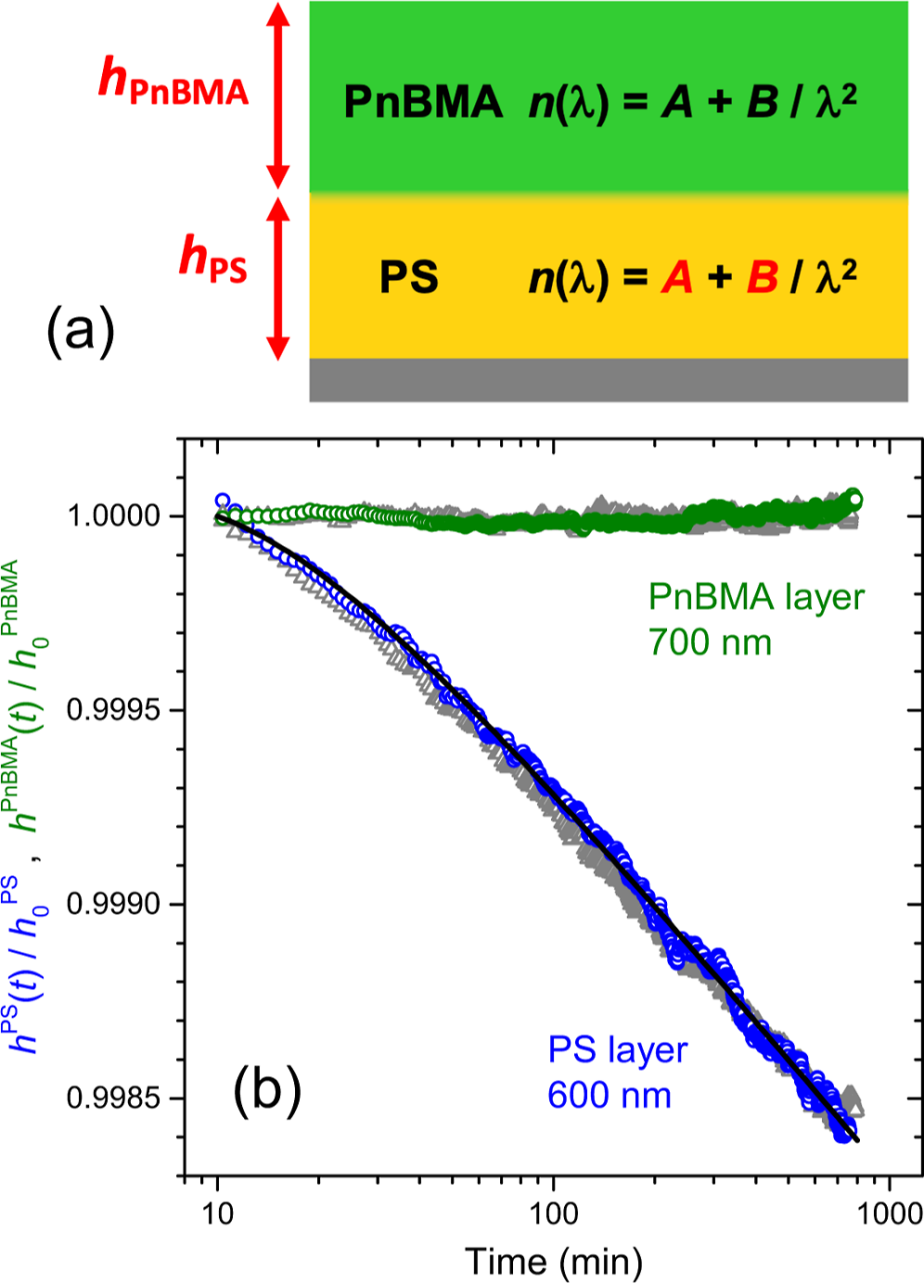
(a) Schematic of the ellipsometer optical layer model used for the PnBMA/PS bilayer films supported on silicon. The four parameters fit are highlighted in red. (b) Graph showing the normalized layer thickness for PnBMA *h*_PnBMA_(*t*)/*h*_0_^PnBMA^ and PS *h*_PS_(*t*)/*h*_0_^PS^ determined from the optical layer model fitting plotted as a function of log time following a rapid thermal quench to the aging temperature of 65 °C. Data for two different samples (colored and gray) are shown to demonstrate reproducibility. The black curve is a fit to eq 1.

A schematic of the ellipsometry layer model used for the present bilayer measurements is depicted in Figure 3a. Following our previous ellipsometry work on PS/PnBMA bilayer films,^29^ the interfacial width between the PnBMA and PS layers was modeled as a fixed 7 nm intermix layer sandwiched between two Cauchy layers representing the two polymers. In the layer model fitting, the thickness of the PS layer *h*_PS_ and the PS Cauchy layer parameters *A* and *B* are fit as both the thickness and refractive index, *n*(λ) = *A* + *B*/λ^2^, of the PS layer will change as the PS layer densifies due to physical aging. To minimize the number of fit parameters the refractive index parameters *A* and *B* of the PnBMA layer were held fixed, as was done previously.^29^ In contrast to our previous work where the physical aging response was only measured for 360 min, these longer 800–1000 min aging runs can result in some small observable variation in the PnBMA layer thickness with time as PnBMA is sensitive to environmental factors such as humidity. As such, we have opted to also fit the PnBMA layer thickness *h*_PnBMA_ in the ellipsometer layer model. Overall, the longer aging runs provide a more complete characterization of the physical aging response of the glassy PS layer with larger total decrease in PS layer thickness due to physical aging and reduced noise.

Figure 3b starts by demonstrating the physical aging response of bulk 600 nm PS layers capped with bulk 700 nm PnBMA layers. The normalized layer thicknesses *h*_PnBMA_(*t*)/*h*_0_^PnBMA^ and *h*_PS_(*t*)/*h*_0_^PS^ determined from the optical layer model fitting to the ellipsometry Ψ(λ) and Δ(λ) data collected from the PnBMA/PS bilayer films are graphed as a function of the logarithm of time. Data from two different samples are shown, one colored and the other in gray to demonstrate reproducibility. As expected, the PS layer exhibits a decrease in thickness approximately linear in log time characteristic of physical aging, while the PnBMA layer thickness remains constant. Following our recent physical aging study on single layer PS films, we can fit the physical aging response of the PS layer to the following equation^7^
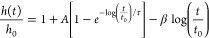
1where β is the physical aging rate, the parameters τ and *A* describe the small curvature at the start of the physical aging run, and *t*_0_ = 10 min corresponds to the initial normalization time. The fit to the data shown in Figure 3 gives a physical aging rate of β = 10.3 × 10^–4^ with *A* = 3.6 × 10^–4^ and τ = 0.49. These values are in good agreement with the bulk aging rate of single layer PS films (for example, the blue data shown in Figure 2 has a best fit value of β = 9.7 × 10^–4^ with *A* = 2.1 × 10^–4^ and τ = 0.22), as well as results from our previous work^7^ that reported a bulk aging rate at 65 °C of β = 10.4 × 10^–4^ with *A* = 2.9 × 10^–4^ and τ = 0.4. With the physical aging response of bulk PS layers within PnBMA/PS bilayer films established, we now decrease the PS layer thickness.

The major challenge with performing physical aging measurements on PnBMA/PS bilayer samples is to meaningfully and accurately extract the small time-dependent changes in the PS layer thickness *h*_PS_(*t*) associated with physical aging. The ellipsometer measures the change in polarization Ψ(λ) and Δ(λ) upon reflection associated with the total PnBMA/PS/SiO_*x*_–Si sample. An accurate optical layer model for the sample is then used to fit the PS layer thickness *h*_PS_. This is challenging, but doable for thick enough layers as the difference in refractive index between the PnBMA and PS layers is 0.1, providing enough optical contrast between the PnBMA and PS layers when they are thick enough. However, as the individual layer and total sample thicknesses, *h*_total_ = *h*_PnBMA_ + *h*_PS_, become much less than the wavelength of light λ ≈500 nm there will come a point when there is no longer enough optical dispersion occurring within the thin layers to accurately extract the PS layer thickness. The aim of the present study is to determine at what PnBMA and PS layer thicknesses this limit occurs and whether we are able to identify any change in the PS layer physical aging response due to the PnBMA/PS interface altering the local dynamics and physical aging response of the PS layer at these thinnest layers.

To obtain a good measurement, the second challenge is to minimize the noise and time-dependent variability of the extracted PS layer thickness *h*_PS_(*t*) because the total decrease in *h*_PS_ due to physical aging is only ∼0.15%, as shown in Figures 2 and 3. Unfortunately, PnBMA is hygroscopic such that its thickness can vary with time due to environmental factors. We mitigate this issue by flowing dry nitrogen gas through the sample chamber during the physical aging measurement, following the same procedure we have previously used.^29^ The temperature stability of the Instec HSC 302 temperature stage is better than 0.1 K. With these experimental protocols, we have been able to reduce the time-dependent oscillations of the PnBMA layer to ∼0.025%. This is excellent, and percentage-wise six times smaller than the anticipated physical aging response. However, if the PnBMA layer thickness is much larger than the PS layer thickness, then such a small variation in the PnBMA layer can still represent a substantial change in total film thickness in comparison to the thickness decrease in the PS layer occurring from physical aging. For example, consider a bilayer sample with *h*_PnBMA_ = 400 nm and *h*_PS_ = 100 nm. A variability in PnBMA layer thickness δ*h*_PnBMA_ of 0.025% corresponds to a total film thickness change of 0.1 nm. In comparison, the total anticipated PS layer thickness decrease for *h*_PS_ = 100 nm, assuming a bulk physical aging rate, would be 0.15 nm. To some extent, we can correct for small artificial variations in the PS layer thickness δ*h*_PS_(*t*) caused by small oscillations in the PnBMA layer thickness δ*h*_PnBMA_(*t*) by knowing that on average the PnBMA thickness remains constant with time. In the absence of physical aging, the total film thickness (*h*_PS_ + *h*_PnBMA_) would be constant meaning a small positive deviation in the PnBMA layer thickness would cause a small negative deviation in the PS layer thickness of equivalent magnitude: δ*h*_PnBMA_(*t*) = −δ*h*_PS_(*t*). This artificial change in the PS layer thickness can be subtracted off leaving behind only the time-dependent change in the PS layer resulting from physical aging. This correction was not required for all data sets, but it did improve the quality of some data sets where the PnBMA layer thickness was particularly variable and the PS layer exhibited a matching unphysical trend. Multiple runs on nominally identical samples were always performed for the different layer thicknesses to ensure we are obtaining reliable and reproducible results that are not simply some artifact of the data fitting or analysis procedure. Corrected data sets were only used if the results agreed with other nominally identical samples with uncorrected data sets. Overall, these changes in data collection, fitting, and analysis have allowed us to achieve reliable results for smaller layer thicknesses. Regardless, as we decrease the PS layer thickness, we will need to also correspondingly decrease the PnBMA layer thickness. We have done this in stages by comparing batches of samples that decrease one layer thickness at a time.

**Figure 4:**
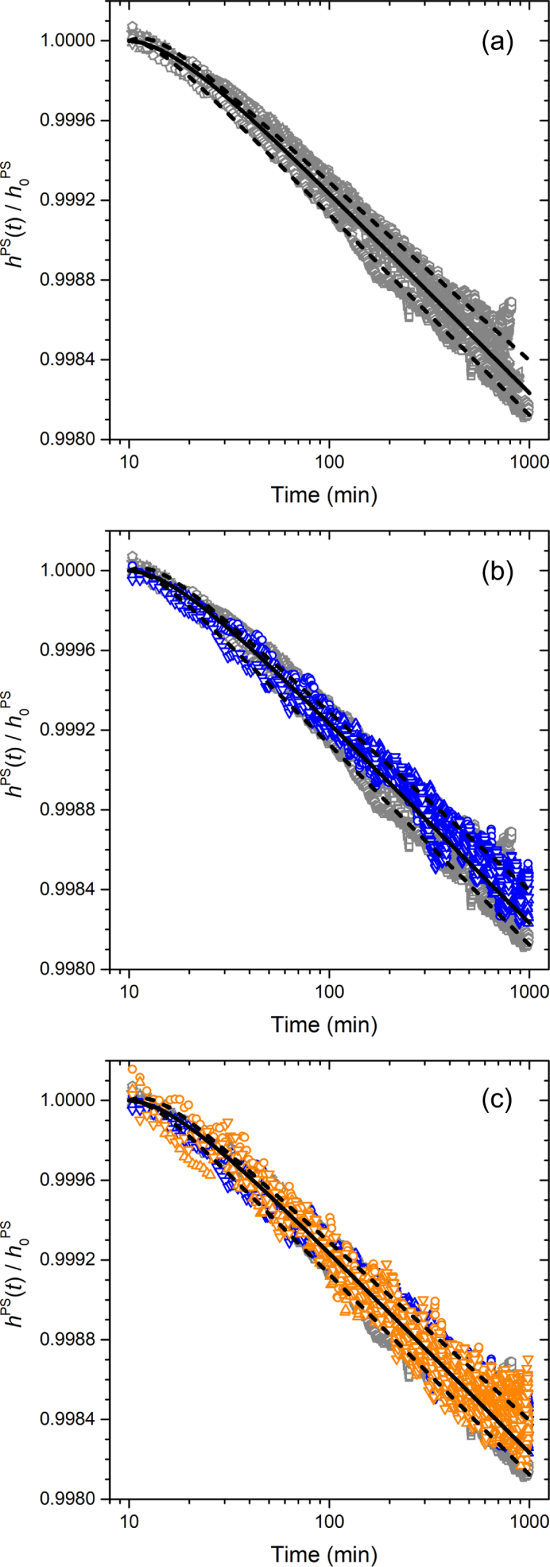
Physical aging response of PS layer thickness normalized as *h*_PS_(*t*)/*h*_0_^PS^ plotted versus log aging time. A series of different batches of PnBMA/PS bilayer samples are compared with varying layer thicknesses: (a) 400–700 nm PnBMA/300–600 nm PS (gray data), (b) 400 nm PnBMA/250–100 nm PS (blue data), and (c) 150 nm PnBMA/75 nm PS (orange data) corresponding to the thinnest layers that could be reliably measured. The black solid and dashed curves are fits to eq 1 indicating the average and range of the bulk gray data.

Figure 4 shows a series of *h*_PS_(*t*)/*h*_0_^PS^ versus log time graphs for different batches of samples. Figure 4a starts with a series of PnBMA/PS bilayer films where the top PnBMA layer thickness was varied between 700 and 400 nm and the bottom PS layer thickness was varied between 600 and 300 nm. This range of samples were found to exhibit equivalent physical aging behavior of the PS layer that matched, to within experimental sample-to-sample variability, the bulk aging response shown in Figure 3. The range of bulk physical aging response is quantified and demarked by a series of fit curves to eq 1. The solid black curve corresponds to a global fit of all the aging data in this batch of 400–700 nm PnBMA/300–600 nm PS samples resulting in best fit values of β = 10.0 × 10^–4^ with *A* = 2.4 × 10^–4^ and τ = 0.26. The dashed curves are fits to the upper and lower data sets giving a range for the physical aging rate β = 8.9 to 10.1 × 10^–4^, which establishes the range of bulk aging response. The first stage of decreasing PS layer thickness is shown in Figure 4b where the PnBMA layer thickness is held fixed at 400 nm, while the PS layer thickness was decreased from 250 to 100 nm. These data are graphed in blue atop the bulk gray data of Figure 4a to illustrate how the physical aging response of the thinner PS layer is essentially unchanged with this decreased layer thickness. At this stage, it was deemed necessary to decrease the PnBMA layer further in order to reliably measure thinner PS layer thicknesses. Ultimately, the thinnest layer thicknesses we found that could be reliably measured were bilayer samples of 150 nm thick PnBMA layers atop 75 nm thick PS layers. These are samples where both layer thicknesses are now much thinner than the wavelength of light (∼500 nm). The physical aging response for a number of different nominally identical 150 nm PnBMA/75 nm PS bilayer samples are shown in Figure 4c as orange data points atop the gray and blue batches of samples from Figure 4a,b. It is clear from these results that these thin PS layers appear to still exhibit a physical aging response equivalent with bulk PS. Given the extensive fluorescence measurements demonstrating large and long-range perturbations to *T*_g_(*z*) resulting from glassy–rubbery interfaces and specifically PnBMA/PS,^18,20,39−41,48^ it is rather surprising to still observe a bulk physical aging response for such thin layers of PS.

### 3.2 Connecting *T*_g_ and Physical Aging Response in PnBMA/PS Bilayer Films

Literature studies that have measured decreases in the physical aging rate of single-layer polymer thin films with decreasing thickness have been able to be rationalized in terms of local reductions in the glass transition temperature *T*_g_ caused by the presence of the free surface.^5,7,8,19^ For example, Pye et al.^5^ and Frieberg et al.^8^ have shown that the physical aging rate of PS thin films decreases for films ≲100 nm in thickness in a manner that can be reasonably correlated with a parametrization of the local *T*_g_(*z*) decrease in these films emanating from the free surface. In essence, a region near the free surface with reduced local *T*_g_ appears to effectively create a region with reduced physical aging. However, differences in the physical aging rate of thin films with molecular weight have been observed that could not be correlated with any *T*_g_ changes suggesting that physical aging may have some different sensitivity to dynamical gradients.^7^

**Figure 5:**
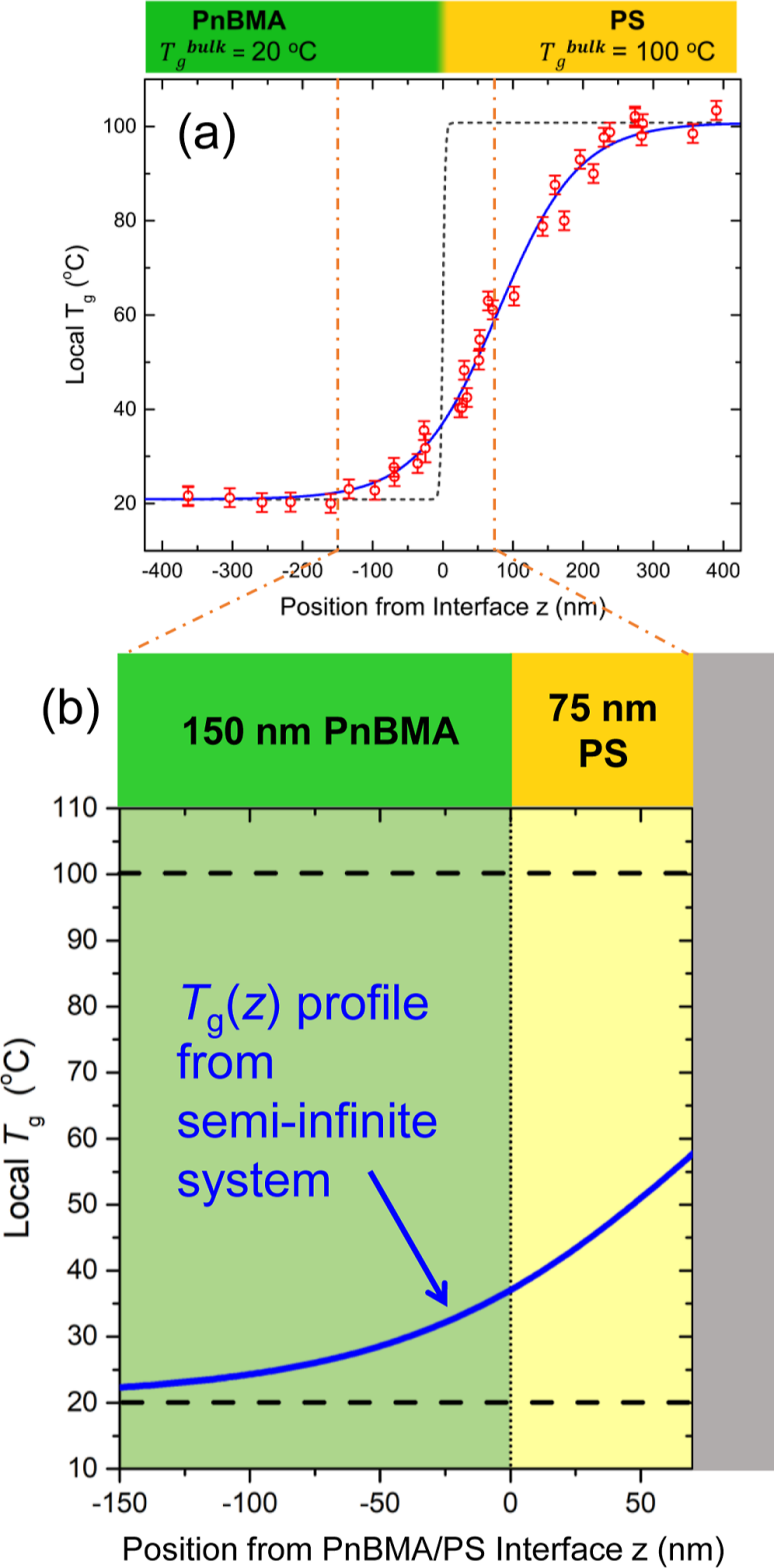
(a) Profile of local glass transition temperature *T*_g_(*z*) across a rubbery–glassy PnBMA/PS interface between two bulk (>450 nm) domains.^20^ (b) Cartoon schematic of a 150 nm PnBMA/75 nm PS bilayer film with the same *T*_g_(*z*) profile from (a) hypothetically superimposed on top assuming the *T*_g_(*z*) profile is unchanged.

For PnBMA/PS interfaces, Baglay and Roth have demonstrated that the local *T*_g_ of PS next to an interface with PnBMA is reduced by ≈60 K, with the local *T*_g_(*z*) perturbation extending ≈250 nm into the PS before bulk *T*_g_ is recovered.^20,39^ The previously measured profile in local *T*_g_(*z*) by Baglay and Roth across a PnBMA/PS interface between semi-infinite domains is shown in Figure 5a.^20^ This is much stronger than the local *T*_g_ reduction of 32 K near a free surface extending into the material to a depth of ≈25 nm that was established by Ellison and Torkelson.^15,18^ As such, one would anticipate a strong decrease in the physical aging rate of PS layers capped with PnBMA beginning at larger thicknesses than is observed for PS films with a free surface. In fact, based on the *T*_g_(*z*) profile shown in Figure 5a, one might even expect that the physical aging rate of the 700 nm PnBMA/600 nm PS bilayer films shown in Figures 3 and 4a would not exhibit a bulk aging response. However, experimentally we observe that these thick PS layers capped with PnBMA have physical aging rates in agreement with bulk single-layer PS films from previous studies^5,7^ and as shown in Figure 2. Previous work has demonstrated that the physical aging response of single-layer PS films is independent of film thickness between 100 and 2500 nm.^5^ This suggests that the PnBMA/PS interface impacts the local dynamics of the glassy PS layer in a manner that is much different compared to a free surface. For the thinner 150 nm PnBMA/75 nm PS bilayer films, a naive mapping of the *T*_g_(*z*) profile from the semi-infinite PnBMA/PS bilayer system to this thinner geometry, as illustrated in Figure 5b, would suggest such a strongly reduced *T*_g_ of the glassy PS layer at effectively all depths from the PnBMA/PS interface that one might anticipate effectively no physical aging at an aging temperature of 65 °C. However, this is contrary to our experimental observation in Figure 4c that the physical aging response of 150 nm PnBMA/75 nm PS bilayer films are equivalent to bulk, implying that this naive mapping is incorrect.

**Figure 6:**
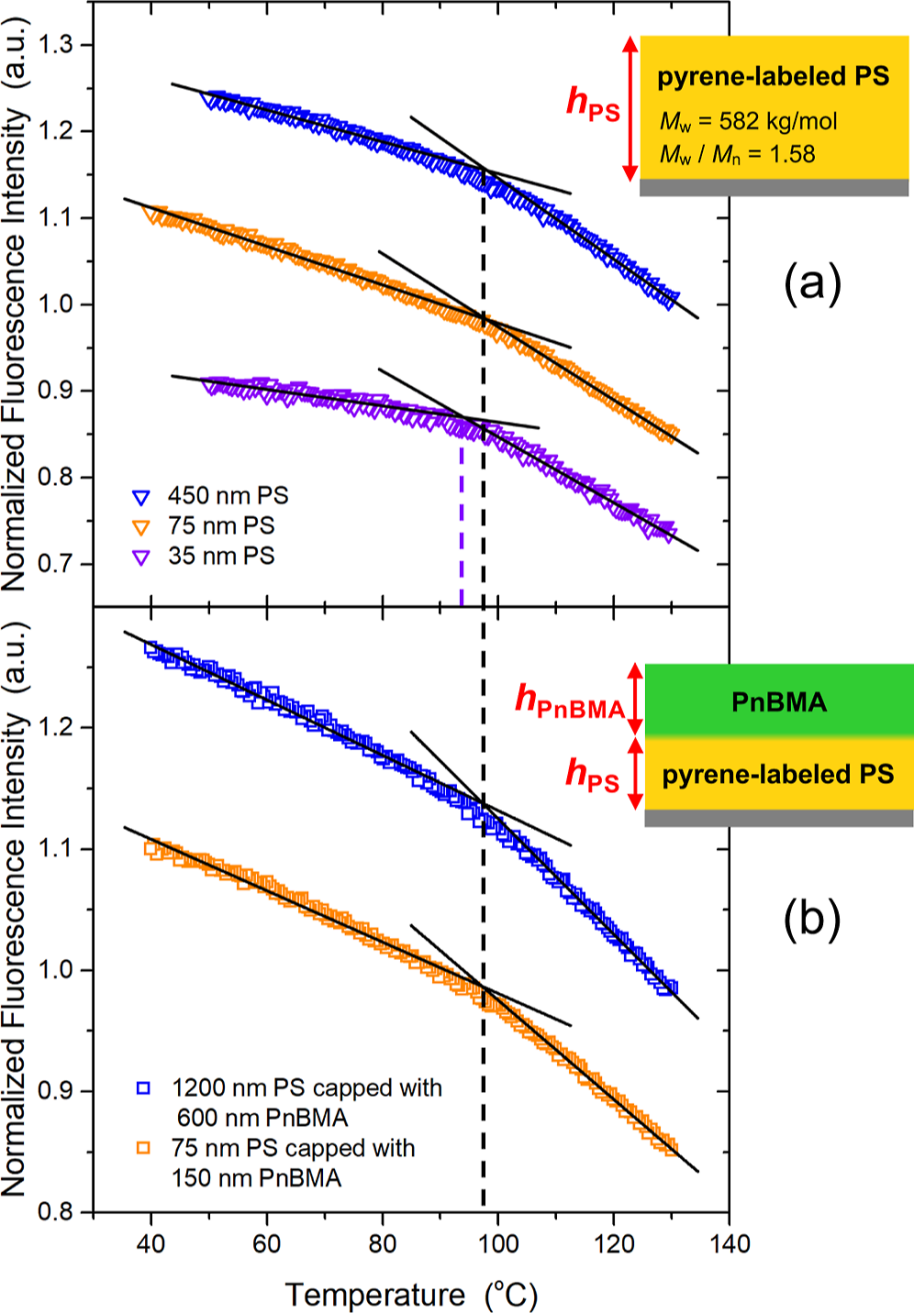
Temperature-dependent fluorescence intensity measurements from pyrene-labeled PS domains for (a) single-layer PS films, giving the average *T*_g_(*h*) of PS films, and (b) PnBMA/PS bilayer films, giving the average *T*_g_(*h*_PS_) of the PS layer.

To better understand this system with finite layer thicknesses, we performed fluorescence measurements on a series of different PS single-layer films and PnBMA/PS bilayer films to compare the impact of the free surface and PnBMA/PS interface on the *T*_g_ values of the glassy PS layer. In Figure 6, we graph the temperature-dependent intensity curves measured from the fluorescence of pyrene labeled to the PS layer, which provides a measure of the average *T*_g_(*h*) of PS single layer films and the average *T*_g_(*h*_PS_) of PS layers of thickness *h*_PS_ capped with PnBMA. The average *T*_g_(*h*) response of single-layer PS films report bulk *T*_g_(*h*) = 98 ± 2 °C for 450 and 75 nm thick PS films, while the *T*_g_(*h*) is reduced to 94 ± 2 °C for 35 nm thick films, as expected from the extensive literature of such measurements.^15,45,49−53^ For the PnBMA/PS bilayer films, bulk *T*_g_ was established with samples consisting of 600 nm PnBMA atop 1200 nm PS layers that reported *T*_g_(*h*_PS_) values of 97 ± 2 °C. When thinner samples of 150 nm PnBMA atop 75 nm of PS were measured, the *T*_g_(*h*_PS_) was also found to be 97 ± 2 °C, unchanged from bulk. Obviously this explains why the physical aging response of the PS layers in the 150 nm PnBMA/75 nm PS bilayer samples shown in Figure 4c are equivalent to bulk.

However, why would such thin PnBMA/PS bilayer films report bulk properties given the previously observed *T*_g_(*z*) profiles across the PnBMA/PS interface shown in Figure 5a? The broad *T*_g_(*z*) profile shown in Figure 5a is that measured for a single PnBMA/PS interface when both the PnBMA and PS domains were semi-infinite in extent, at least 450 nm or greater.^20^ In such a geometry, the *T*_g_(*z*) gradient was able to extend as far as the material required to recover bulk behavior, unimpeded by another interface or finite size of the material. In 2017, Baglay and Roth showed that a finite domain size alters the behavior of the *T*_g_(*z*) gradient.^48^ The *T*_g_(*z*) profile inside a 300 nm PS domain sandwiched between two PnBMA domains was measured by local pyrene fluorescence. In such a PnBMA/PS/PnBMA trilayer geometry, the behavior of the 300 nm PS domain would be perturbed from PnBMA/PS interfaces on both sides. The measured *T*_g_(*z*) profile was found to not simply be a linear superposition of Δ*T*_g_ changes from both interfaces indicating that some other factor associated with the finite size of the domain was also altering the gradient in dynamics.^18,48^ This is reminiscent of the observation by Ellison and Torkelson that the local *T*_g_ at the free surface of PS thin films changes when the total film thickness becomes less than 60 nm.^15^ Counterintuitively, they showed that the local *T*_g_ at the free surface of PS becomes less reduced from bulk for thinner films. A PS film of total thickness of ≈25 nm has a local *T*_g_ near the free surface reduced by only 15 K from bulk compared to films >60 nm that show a local *T*_g_ reduced by 32 K.^15^ Thinner films appear to have a more homogeneous distribution in local *T*_g_, from which Ellison and Torkelson suggested that perhaps the size of the glassy domain needed a certain extent to support a strong gradient in dynamics. Similarly, Merrill et al. have recently shown that very thin films of PS with *h* < 20 nm exhibit a reduced glass transition breadth with decreasing film thickness suggesting that the dynamics of very thin films become more homogeneous.^53^ And yet, Christie et al. have shown that PnBMA–PMMA diblock copolymers self-assembled into lamellar geometries have local *T*_g_(*z*) profiles that vary by ≈70 K within a span of only 8 nm.^54,55^ There is clearly an important need to understand how dynamical gradients and local *T*_g_ profiles are altered in systems with varying domain size.^18,56^

In addition, it is well recognized that different experimental techniques capture different aspects of the altered dynamics and material properties with decreasing film thickness. For example, Paeng and Ediger have observed two distinct dynamical populations in dye reorientation times for thin polymer films, where even for the thinnest films some fraction of the dynamics still exhibit bulk-like dynamics.^57,58^ In contrast, pyrene dye’s measure of *T*_g_ samples only one aspect of the dynamics, which has been previously associated with the faster portion of the distribution.^59^ Physical aging measures the time-dependent volume contraction of the glass that would naturally be limited by the slowest portion of the dynamical distribution. Our physical aging results on thicker PnBMA/PS bilayer films find that the distribution of relaxation times in the glassy PS layer is predominantly bulk like, even for samples where *T*_g_(*z*) measurements by pyrene dye have reported large and long-range reductions.^20^ This result is consistent with recent dye reorientation measurements by Paeng and co-workers in PS near glassy–rubbery polymer interfaces that reported bulk dynamics.^60^ For the thinner PnBMA/PS bilayer films, the pyrene dye *T*_g_(*h*) measurements presented in Figure 6 find that the average *T*_g_ is equivalent to bulk for the 150 nm PnBMA/75 nm PS bilayer samples, which is consistent with the physical aging behavior. This suggests that the large and long-range *T*_g_(*z*) profile for the thicker layer films, illustrated in Figure 5, is altered in the thinner layer films by the finite size of the domains. Future work will focus on mapping the *T*_g_(*z*) profile in PnBMA/PS bilayer films, for example specifically 75 nm PS layers capped with PnBMA, to better understand how the dynamical gradient is reshaped by the limited range of the domain.

Recently, Gagnon et al. proposed that the long-range *T*_g_(*z*) gradients observed across glassy–rubbery polymer domains may result from the ability of acoustic waves to propagate across a broadened polymer–polymer interface with interfacial width ≈5 nm.^61^ Specifically, acoustic waves with wavelengths λ ∼5 nm that are part of the vibrational density of states near the boson peak would be expected to propagate across such a broadened interface providing a long-range mechanism by which acoustic (density) waves from the rubbery domain could effectively trigger density fluctuations in the glassy domain leading to α-relaxations. These acoustic waves have energies comparable to those associated with the boson peak which are thought to be precursors of α-relaxations.^62−66^ It is possible that acoustic waves from the rubbery PnBMA domain travel into the glassy PS domain resulting in density fluctuations that effectively lower *T*_g_ as measured by pyrene fluorescence, while slower domains still persist limiting the structural relaxation associated with volumetric physical aging decay to bulk time scales. The vibrational density of states are known to depend on the overall size of the system as acoustic waves will reflect at the sharp interfaces of the boundary, as such this type of mechanism would be expected to depend on the finite domain size of the material. There is clearly a need to investigate these glassy–rubbery systems with different experimental techniques to better understand the extent of the resulting property changes and their fundamental origins.

## 4 Conclusions

In this work, we have investigated the ability of ellipsometry to resolve the physical aging response of ultrathin glassy PS layers from PnBMA/PS bilayer films. As physical aging involves the slow densification of an amorphous glassy state on a logarithmic time scale, it is necessary to fit both the small decrease in PS layer thickness *h*_PS_ and corresponding small increase in refractive index *n*(λ) that occur with time. Data quality was also improved by fitting the PnBMA layer thickness *h*_PnBMA_ to accommodate small oscillations in its thickness from environmental factors. For ultrathin layer thicknesses that are substantially less than the wavelength of light (∼500 nm), obtaining enough dispersion of the light through the sample eventually limits the minimum layer thicknesses that can be adequately resolved from PnBMA/PS bilayer films where the refractive index contrast Δ*n* is only 0.1. We determined that the minimum PnBMA/PS layer thicknesses that could be adequately measured to properly resolve the physical aging response of the glassy PS layer was 150 nm of PnBMA atop 75 nm of PS. Surprisingly, we observed that 150 nm PnBMA/75 nm PS bilayer films still report a bulk physical aging response of the glassy PS layer, an unexpected outcome given that previous literature had established that a PnBMA/PS interface causes a larger local *T*_g_ reduction in PS (≈60 K) than that of a free surface (≈30 K).^15,18,20^

The magnitude of these local *T*_g_ reductions, however, were established for semi-infinite PS layer thicknesses where the depth of the dynamical gradient can extend as far as the amorphous glassy material would like. In contrast, within the finite layer thickness of 75 nm for the glassy PS layer, the dynamical gradient is spatially constrained, sandwiched between the soft rubbery–glassy PnBMA/PS interface and the hard boundary of the SiO_*x*_–Si substrate. The impact of finite spatial constraint on the breadth of dynamical gradients is an important open question at present with only a handful of existing studies providing some insight.^15,18,48,53,54^ To better understand our unexpected physical aging observation in ultrathin 150 nm PnBMA/75 nm PS bilayer films, we also performed fluorescence measurements to determine the average glass transition of PS layers of varying thickness. These results demonstrated that 75 nm PS layers in PnBMA/PS bilayer films had *T*_g_(*h*_PS_) values equivalent to bulk, consistent with the observed physical aging response. However, this then implies that the finite size of the 75 nm PS domain has strongly altered the *T*_g_(*z*) profile from the previously measured broad extent found for the semi-infinite PnBMA/PS system.^20^ The role of finite size is thus an important puzzle we plan to address in future work.

Overall, we are able to draw two key conclusions from this work. (1) The rubbery–glassy PnBMA/PS interface appears to perturb the properties of the glassy PS domain in a manner different from that of a free surface. Even though long-range *T*_g_(*z*) changes are observed by pyrene fluorescence across PnBMA/PS interfaces, the physical aging measurements report bulk-like behavior of the glassy PS domain. We believe these observations reflect how different experimental techniques are sensitive to different aspects of the dynamical distribution, where physical aging is biased toward the slowest portion and pyrene fluorescence the faster portion. (2) The previously observed large and long-range *T*_g_(*z*) reductions measured by pyrene fluorescence across the PnBMA/PS interface in systems with semi-infinite domains cannot be straightforwardly mapped onto PnBMA/PS bilayer films with thinner layers. The magnitude and extent of the *T*_g_(*z*) gradient appears to be altered by the finite size of the domains.
